# Investigation of color and physicomechanical properties of peek and pekk after storage in a different medium

**DOI:** 10.1038/s41598-024-54695-5

**Published:** 2024-03-04

**Authors:** Nihan Kaya, Rafat Sasany, Nuran Yanıkoglu, Busra Tosun

**Affiliations:** 1 Department of Prosthodontics, Faculty of Dentistry, University of Giresun, Giresun, Turkey; 2 Department of Prosthodontics, Faculty of Dentistry, University of Biruni, İstanbul, Turkey; 3 Department of Prosthodontics, Faculty of Dentistry, University of Ataturk, Erzurum, Turkey; 4 Department of Prosthodontics, Faculty of Dentistry, University of Abant İzzet Baysal, Bolu, Turkey

**Keywords:** Materials science, Optics and photonics

## Abstract

The aim of this study is to assess color stability, solubility, and water sorption on polyether ether ketone (PEEK) and polyether ketone ketone (PEKK) after immersion in different storage conditions. Material and Methods Ninety disc-shaped specimens (8 × 2) were obtained from CAD/CAM blocks [PEEK (n = 45) and PEKK (n = 45)]. Before immersion, baseline color value data were recorded with a spectrophotometer. The specimens were soaked in three solutions red wine, coffee, and distilled water at 37 °C for 28 days. Following immersion, color values were remeasured, and color-change values (ΔE) were calculated. Water sorption and solubility were assessed by mass gain or loss after storage in water for 28 days. The Kruskal–Wallis and the Mann–Whitney U test were used for analysis (P = 0.05). Results ΔE00 between PEEK and PEKK was significantly different statistically (P < 0.001). PEEK presented higher water sorption than PEKK (P = 0.005). The difference in solubility between PEEK and PEKK was not statistically significant (P = 0.163). The materials and storage medium types had a statistically significant impact (P = 0.100). In terms of staining potential, the solutions tested in this experiment were ranked as: coffee > red wine > distilled water. The results of this study demonstrated that PEKK was more successful in polymer-containing CAD/CAM materials as it exhibited less color change and water absorption.

## Introduction

The development of computer-aided design and manufacturing (CAD-CAM) technology has resulted in the introduction of novel materials that can be precisely machined to produce dental prosthetics^1,2^. Today, polyether ether ketone (PEEK) and polyether ketone ketone (PEKK), subordinate members of the polyaryletherketone (PAEK) group, have been promoted for dental implants, temporary implant abutments, removable prostheses, fixed partial dentures, implant healing caps, and implant-supported hybrid prostheses^3–16^. The PEKK is a methacrylate-free thermoplastic high-performance material^9^ and increasing biomaterial with properties suitable for dental and medical applications. PEEK and PEKK materials are available in colors comparable to those of teeth, including dentine, white, and universal^8–11^.

Functional characteristics include fine polishing ability, low water sorption, slight polymerization shrinkage, low residual monomer content, and color stability^12^, thus offering excellent promise for a variety of dental applications, such as dental implants, implant-supported fixed prostheses, crowns, bridges, endoposts, denture framework, and tooth restorations^8–16^. An important parameter for patient satisfaction and compliance is the color stability of the material and a natural color appearance of dental prostheses. Critical factors in color stability include restoration polishing, material composition, patient dietary preferences, and cleaning routines^17^.

The fundamental characteristics of a dental prosthesis, such as the kind of resin matrix, the size and proportion of the filler, and filler distribution, affect potential discoloration. The color of dental restorations may change due to intrinsic factors such as the resin matrix, percentage of filler, filler distribution or composition, polymerization of the restorative material, and restoration manufacturing technique^17–21^ or extrinsic factors such as staining from caffeine, theobromine, anthocyanidins, tannins, and nicotine in drinks, mouthwashes, and smoking^22^.

Polymers can absorb water and various chemicals from the oral environment^23^. This absorption may cause dissolution of the restoration surface and thus weight loss^24^. Water absorption and solubility affect the color stability of the restorative material as well as adversely affect its mechanical properties such as strength and abrasion resistance^23,24^. This adverse effect on mechanical properties may reduce the clinical life of restorative materials^23,24^. In addition to the mechanical properties of prosthetic materials, color stability plays a very important role in clinical success. Depending on the oral hygiene, nutrition and smoking habits of the patients, the color change rates of the restorations may differ. As a result, these discolorations can result in dissatisfaction with the restorations^25,26^.

However, water sorbtion, solubility, and discoloration of high performance polymers have not been thoroughly investigated. The objective of the study was to compare how monolithic crowns constructed of PEKK and PEEK changed color, solubility, and water sorption over time. The null hypothesis was that there would not be a discernible difference in color change, solubility, and water sorption between PEEK and PEKK following different storage conditions.

## Material and methods

Prior to the study, approval (No. of approval: 03/2023/10) was obtained from the Ethics Committee of Atatürk University Faculty of Dentistry, Erzurum, Turkey. A statistical power analysis (power = 80, α = 0.05, f = 0.40) performed with the G*Power software program (v3.0.10) determined the total number of samples as n = 90, with six subgroups of n = 15 samples in each group**.**

PEEK (CopraPeek Whitepeaks Dental Solutions GmbH & Co. KG, Wesel, Germany) (A2 light) and PEKK (Pekkton Ivory Cendres + Metaux SA, Switzerland) were examined in this study as two commercially available CAD-CAM translucent materials (A2 light) (Table 1).

**Table 1:** Summary of the materials used in this study.

Materials and equipments	Manufacturer
PEEK	CopraPeek Whitepeaks Dental Solutions GmbH&Co.KG, Wesel, Germany
PEKK	Pekkton Ivory Cendres + Metaux, SA Sweden

Ninety disc-shaped specimens (8 × 2 mm) were prepared in a cutting machine (Buehler series 15 LC diamond; Illinois Tool Works Inc,) under running water in a special dental prosthesis laboratory. Each specimen’s surface was polished at P600, P800, and P1200^27,28^, and then cleaned in an ultrasonic for 5 min. The specimens were divided into three subgroups for each material. Each specimen’s color, water sorption, and solubility values were recorded before and after immersion in the solutions. All materials used are summarized in a clinical spectrophotometer (VITA EasyShade V, VITA Zahnfabrik) was used to measure the color using the CIEDE2000 color coordinates in “tooth single” mode before being exposed to one of the solutions^29,30^. The spectrophotometer had an 8 mm aperture. Under D65 standard illumination in a 00 observation and a 450 light source, the specimens’ colors were measured^30^. On a neutral gray background, measurements were assessed 3 times for each item at the same time of day. After the initial measurements, the specimens were stored in one of the three solutions for 28 days, a storage time representing 2.5 years of clinical service^31^.

Group 1: 300 mL distilled water (pH: 6.47) was designated a control group.

Group 2: 3 g of coffee (pH: 5.50) (Nescafe Classic) dissolved in 300 mL of boiling distilled water.

Group 3: 300 mL red wine (pH: 3.28) (DLC Öküzgözü 2009, Doluca, Istanbul, Turkey). The staining solutions were kept in a dark environment (at 37 ± 2 °C) and changed every 2 days^32^.

The specimens were cleaned by brushing their surfaces under running water after each storage period. After 10 min of ultrasonic cleaning in distilled water, the samples were dried. Color coordinate measurements were repeated for all specimens.

The color difference values resulting from the experimental conditions were calculated with CIEDE2000 using the following formula^33^:

$$\Delta E_{00} = \sqrt {\left( {\frac{\Delta L}{{K_{L} S_{L} }}} \right)^{2} + \left( {\frac{\Delta C}{{K_{C} S_{C} }}} \right)^{2} + \left( {\frac{\Delta H}{{K_{H} S_{H} }}} \right)^{2} + R_{T} \left( {\frac{\Delta C}{{K_{C} S_{C} }}} \right)\left( {\frac{\Delta H}{{K_{H} S_{H} }}} \right)}$$

The parametric factors of the CIEDE2000 color difference formula were set to 1^34^. ΔE_00_ ≤ 1.30, which is ranked as perceptibility, and ΔE_00_ > 2.25 is the acceptability threshold^35^.

The samples were weighed repeatedly at 24-h intervals until they reached their constant weight. The values were taken with a precision balance (GH-252, A&N Company, Japan) (M1). The samples were then incubated for 28 days in a 37 ºC incubator in sealed bottles containing 2 mL of distilled water (pH 7.2). After the storage period, the samples were washed under running water, dried with liquid-absorbent papers, and weighed again (M2). Samples were then stored in a silica gel-containing desiccator at 37 ºC to remove the absorbed water and were weighed daily until a constant mass was reached (M3).

M1: Dry weight of samples (mg)

M2: The mass of the sample after immersion in distilled water for 28 days (mg)

M3: The mass of the sample after being conditioned in a desiccator with silica gel (mg)

V: Volume of samples (mm^3^)

The water sorption (WS) and solubility (sol) for 28 days of storage in water were calculated using the following formulas:

$${\text{WS}}:{\text{ M2}}{-}{\text{M1}}/{\text{V}}\quad {\text{Sol}}:{\text{ M1}}{-}{\text{M3}}/{\text{V}}$$

The statistical analysis application IBM SPSS 20 was used to conduct the analyses. The significance of the differences in solubility, water sorption, and color change between the two materials was evaluated using the Kruskal–Wallis test. The two that differed from the others were determined using Conover Multiple Comparison tests when the *p* values from the Kruskal–Wallis test results were statistically significant. The Mann–Whitney *U* test was used to assess the significance of the color change, solubility, and water sorption differences between the materials within two independent groups (p = 0.05).

## Results

**Figure 1:**
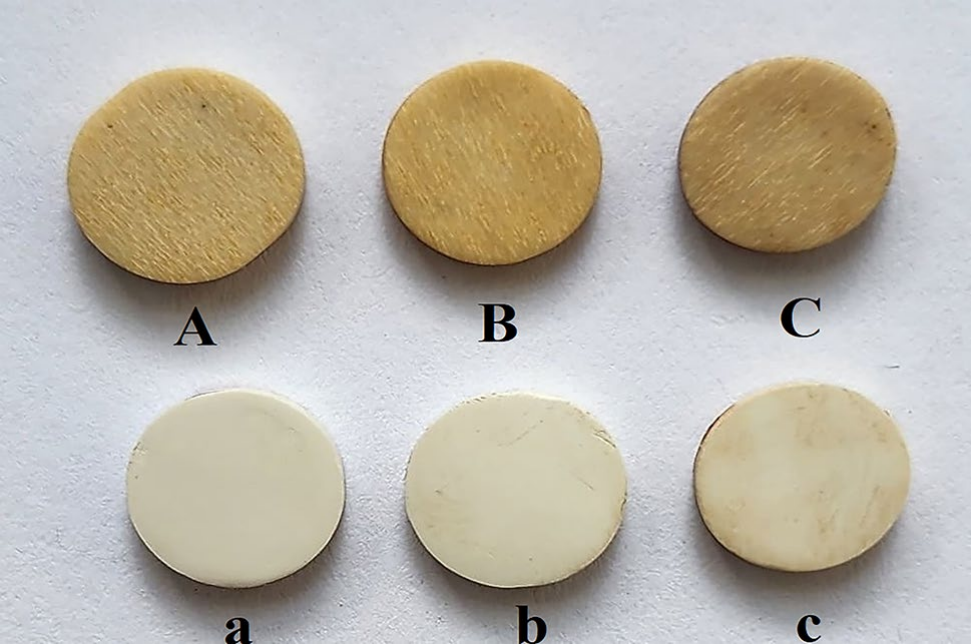
Images of samples: capital letters indicate PEEK after storage in distilled water, red wine, and coffee, respectively. The lowercase letters showed PEKK after being stored in distilled water, red wine, and coffee, respectively.

**Figure 2:**
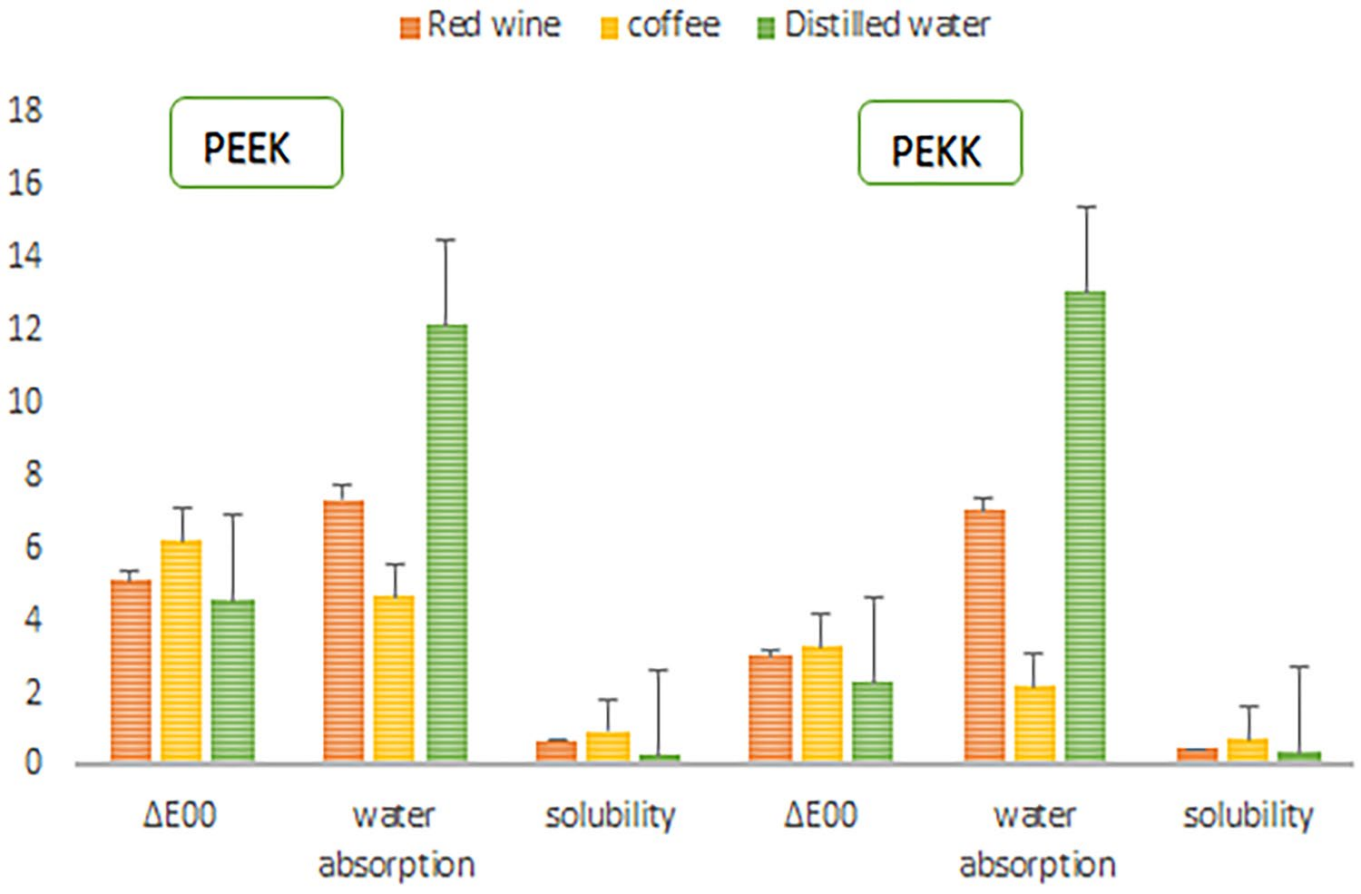
Means values of ∆E_00,_ water sorption, and solubility with storage medium results divided into each material. Error bars represent standard deviations.

While examination of the color changes manifested significant differences between PEEK and PEKK (Table 2) only in PEEK material was a statistically significant difference observed between different storage media (*P* = 0.001) (Table 3). The highest level of discoloration in PEEK was observed in samples immersed in coffee(P = 0.002), followed by red wine (P = 0.004) and distilled water samples(P = 0.004)(Fig. 1). In the PEKK material, there was no significant difference in the samples exposed to red wine and coffee when compared to the control group (P = 0.359) (Table 3). The amount of water sorption in the PEEK material was recorded as significantly higher statistically than in PEKK (P = 005) (Table 2). All restorative materials used in this study showed color changes above the clinically acceptable level in all solutions. In both material groups, the amount of water sorption was observed in the samples kept in distilled water, red wine, and coffee from high to low in terms of storage media (Table 3). There was no statistically significant difference in terms of solubility between materials and incubation media (P = 0.163) (Table 2). The mean values ∆E00, water sorption, and solubility are given in Fig. 2.

**Table 2:** Descriptive statistics of color change, water sorption, and solubility of each material separately.

	PEEK	PEKK	P
∆E00	3.94 ± 0.16	1.78 ± 0.94	< 0.001
Water Sorption	3.40 ± 0.34	2 ± 0.61	0.005
Solubility	0.31 ± 0.9	0.2 ± 0.8	0.163

**Table 3:** Descriptive statistics of color change, water sorption, and solubility after different media for each material separately.

		Red wine	Coffee	Distiled water	P
PEEK	∆E00	3.40 ± 0.34	4.14 ± 0.36	3.04 ± 0.56	0.003
	Water sorption	2.35 ± 0.19	1.56 ± 0.68	4.53 ± 0.37	0.039
	Solubility	0.68 ± 0.33	0.98 ± 0.74	0.28 ± 3.78	0.087
PEKK	∆E00	2.00 ± 1.2	2.20 ± 0.7	1.52 ± 0.48	0.359
	Water sorption	2.45 ± 0.17	0.73 ± 0.52	4.04 ± 0.38	0.001
	Solubility	0.47 ± 0.82	0.73 ± 1.55	0.38 ± 1.07	0.323

## Discussion

The null hypothesis was rejected because this study found statistically significant differences between PEEK and PEKK in color change and water sorption. The PAEK family, comprised of thermoplastic polymers with high biocompatibility and good mechanical properties, is widely used in many areas of dentistry^3–16^. However, the discoloration of aesthetic restorations poses a major problem. No previous studies have compared color changes caused by beverages in PEEK and PEKK. However, some studies have looked into color variations between PEEK, composite, or PMMA. One study examined the color change in PEEK, PMMA, and composite materials by soaking them in four test media, and concluded that the lowest color change was in PEEK^17^. In another study, polyoxymethylene and PEEK materials were kept in water, wine, coffee, cleanser, and a combo bath. They concluded that the color change in the PEEK material was lower. Furthermore, among the solutions tested, the amount of color change in the samples kept in coffee was highest^36^. Similarly, in the present study, among the various storage media, the greatest color change in PEEK materials was observed in samples stored in coffee, and this discoloration was greater than in PEKK materials. Such a potent effect of coffee has also been found in studies on indirect restorative and denture base resins^22,37^ because coffee contains many high molecular weight water-soluble colorants.

The effect of wine on the color of the materials was less than that of coffee. Due to its low ethanol content and dense structure, wine does not adhere to the surfaces of the materials. However, the change in the polymeric pigment level of the wine, since grape composition can be affected by factors such as fermentation and vintage, may produce different results on color changes of the materials^38^.

In the current study, the color change in PEEK material of the immersing solutions was significantly different statistically, while this difference was not significant in the PEKK material. In comparison to PEEK, PEKK features a second ketone group that boosts color stability^10,13^. It was thought that the additional ketone group and materials in PEKK could cause this difference.

Most polymers absorb more or less water, but not all exhibit changes in properties. According to several authors, only water that forms hydrogen bonds with macromolecules modifies their properties^6,7^. Strongly polar polymer chains can use hydrogen bridges to bind water. The ketone group (C 14 O), the sulphone group (O 14 S 14 O), and nitrogen are a few examples. Oxygen on either side of a ketone group prevents hydrogen bonds from forming^6^. One such is PEKK, which has ketone groups surrounded by oxygen^7^. PEEK and PEKK feature aromatic rings; however, the proportion of ether- to keto-groups varies^13^. There are some differences between PEKK and PEEK. PEKK has a second ketone group, and it increases polarity and backbone rigidity, which results in an increase in glass transition and melting temperature^13^. Moreover, PEKK displays both amorphous and crystalline behavior, and different products can be produced. A PEKK with 60% straight and 40% kinked segments melts at 305 °C but PEEK with 80% straight and 20% kinked melts at 360 °C. In addition, the extra ketone group in PEKK has strong polymer chains and shows better physical and mechanical properties, such as compressive strength**. **They are made of an elastic material that is resistant to breakage and has good shock absorption^16^. The additional ketone group in PEKK also possesses strong polymer chains and exhibits superior physical and mechanical qualities, such as compressive strength^15,17^. In the current study, the water absorption in PEKK material was found to be lower than in PEEK, which is in line with existing literature^17^.

There was no statistically significant difference in solubility between the materials and storage media in the present study. Perhaps the explanation is that the more homogenous a polymer is, the fewer soluble properties it has and the less water it absorbs^14^. Industrially produced materials have a lower risk of porosity and consequently higher and more solid mechanical characteristics, which was supported by this investigation^5^. Another study found that storing micro hybrid composite resin in water, alcohol, and bacterial acid had a negative impact on the surface qualities^3^.

This study has its limitations. It is acknowledged that polymers may be susceptible to water sorption, which could decrease their color stability. The narrow window of the spectrophotometer may cause edge loss, affecting the measurements’ accuracy. In addition, the testing was done at one time. Testing at several intervals may provide more accurate results. In this in vitro study, after soaking in different solutions, the PEEK material was observed to discolor beyond the clinically acceptable level, while the color change in the PEKK material was only perceptible in all environments. Again, in terms of water absorption, PEKK was found to be more successful by absorbing less water than the PEEK material; however, the oral environment could not be fully simulated. We recommend that clinical studies are needed to detect color changes in polymer-containing CAD/CAM materials after soaking in different storage media.

## Conclusions

Within the limitations of this study, the following conclusions were drawn: significant differences were observed between the materials in terms of color change and water sorption. Color change and water sorption values were higher in the PEEK samples than in the PEKK samples.While PEEK showed color change exceeding the acceptable level in all samples, in PEKK, the color change was ranked perceptible. In PEEK and PEKK CAD/CAM blocks, samples kept in coffee showed the highest color change values, followed by red wine and distilled water, in that order. Different storage media caused significant differences between PEEK and PEKK in terms of water sorption. In both materials, the highest water sorption values were observed in distilled water, followed by red wine and finally coffee.The results of this study demonstrated that PEKK was more successful in polymer-containing CAD/CAD materials as it exhibited less color change and water absorption.

## Data Availability

The data will be made available by the corresponding author upon reasonable request.
